# Drug use and harm reduction in Afghanistan

**DOI:** 10.1186/1477-7517-2-13

**Published:** 2005-09-07

**Authors:** Catherine S Todd, Naqibullah Safi, Steffanie A Strathdee

**Affiliations:** 1 Division of International Health & Cross-Cultural Medicine, Department of Family & Preventive Medicine, University of California, San Diego, 9500 Gilman Drive, 0622 La Jolla, CA, USA, 92093-0622; 2 National HIV/AIDS Control Program, Ministry of Public Health, Massoud Road, Kabul, Afghanistan

## Abstract

Opium has been cultivated in Afghanistan since 1100 A.D., although production has steadily increased since 1979. Currently, Afghanistan produces three-quarters of the global opium supply, with injection drug use and HIV currently following the opium trade route through Central Asia. Although systematic studies are lacking, heroin use appears to be on the rise in Afghanistan. The purpose of this paper is to briefly provide historical background and current statistics for drug production and use in Afghanistan, to discuss the new government's policies towards problem drug use and available rehabilitation programs, and to assess Afghan harm reduction needs with consideration of regional trends.

## Introduction

Afghanistan is at a cross-roads; the country is emerging from more than twenty years of political and social unrest as the leading global producer of opium in a geographic region widely affected by drug use, particularly injection drug use, and blood-borne infections, including human immunodeficiency virus (HIV). Countries bordering Afghanistan (with the exception of Turkmenistan, for which there is no available data) are experiencing concentrated epidemics of HIV and hepatitis C in IDU populations [1-4]. Afghanistan is currently at risk for these potentially destabilizing events. Historically, countries slow to respond or instituting only punitive measures for ascending rates of drug use have experienced dramatic outbreaks of HIV and hepatitis among injection drug users (IDU), often with diffusion into the general population [5-7]. The rationale for this paper is to examine the current situation and policy of Afghanistan, as little is known about substance abuse in this country. We will briefly provide historical background and current statistics for drug production and use in Afghanistan, present the new government's policies towards problem drug use and available rehabilitation programs, and compare the situation in Afghanistan to that of the surrounding geographic region, much of which is experiencing the most rapid increase of HIV cases due to injection drug use.

## Opium History in Afghanistan

We will focus on opium, the substance with greatest impact on risk of blood borne infections in Afghanistan. Information was obtained from electronic searches through PubMed and Google, with additional information obtained through site-specific searches, such as United Nations Office of Drugs and Crime (UNODC). Selected search words were: opium, Afghanistan, trafficking, Central Asia, and heroin. While we have chosen to focus only on opium, the same routes for trafficking opium are used to transport both other illicit substances, such as cannabis/hashish (also produced in Afghanistan) and amphetamines and licit drugs of abuse, such as pharmaceutical compounds (e.g. benzodiazepines, opioid analgesics), and volatile inhalants.

Afghanistan, along with Pakistan and Iran, form the Golden Crescent, an area known for opium and cannabis cultivation and trafficking from the time poppies were introduced from Europe by Arab traders along the Silk Road [8]. Opium production in Afghanistan did not reach large scale until the Russian invasion in 1979. The growth in production was attributed to direct loss of government controls on production and indirect market demand created by decreased production due to political disruption in Vietnam and Laos, formerly the chief suppliers to Europe and North America [9,10]. By this time, Iran had significantly decreased opium production due to blockage of trade routes and severe punishment for drug-related convictions by the new theocratic regime [10]. Restrictions on cultivation and refining in Pakistan in the mid to late 1990's led to the shift of these activities to Afghanistan, resulting in the creation of new trade routes into Pakistan and Central Asia [11]. Opium cultivation was further encouraged by warlord commanders in constant conflict with each other following the Russian retreat in 1989. These commanders required economic support for military actions in response to loss of United States funding to Najibullah's government in 1991 [12]. During the *mujaheddin *[freedom fighters] era, opium and heroin production rose steadily with Afghanistan becoming the leading global supplier, overtaking Burma in the mid-1990's [13]. Since this time, either Afghanistan or Burma have contributed the greatest percentage to the world's opium market, with Afghanistan being the single largest country producer for the last four years.

The rise of the Taliban regime was marked with steadily increasing opium production, despite their pledge to "cleanse Afghanistan of the poisoned poppies" [14]. Increased opium production has been attributed to economic realities faced by the Taliban, who received little external donor support due to international sanctions. The Taliban charged a 10% tax to opium farmers, netting $20 million or more each year, and controlled the opiate trade, with confiscated boxes bearing the words, "Not for use by Muslims" [15,16]. However, in 2000, the Taliban, now controlling the majority of Afghanistan, banned opium cultivation and enforced harsh punitive measures against drug use, which included maiming the hands of drug users. These steps, as well as severe drought in Afghanistan, were highly effective in reducing the amount of available opiates in the world market, resulting in drug shortages in Europe and a ten-fold increase in price [13,17]. Some believe the move was economically motivated to increase price, but this will remain open to debate as the Taliban were deposed in 2001 [18,19].

Within the year following removal of the Taliban regime, opium production recovered to near-record levels, with 3400 and 3600 metric tons produced in 2002 and 2003, respectively. In 2003, total income to opium farmers alone was equal to half of the legal gross domestic product and illustrated that, despite Hamid Karzai's declaration of a *jihad *[holy war] on opium, regional commanders continue to rely on opium production and trafficking to maintain their strongholds [13]. Opium cultivation has been revived in southern provinces and introduced in eastern and northern Afghan provinces, likely due to economic consideration as it is at least twelve times more profitable than wheat [13,20,21]. In 2004, a UNODC survey was performed to assess opium production within Afghanistan [21]. The study reports opium cultivation in all provinces with 2.9% of all arable land devoted to this purpose, though as much as 29% is cultivated in some provinces. The estimated crop for 2004 would have exceeded the record set in 1999 had drought and other plant stressors not compromised crop yields [21]. Despite these losses, Afghanistan produced 87% of the global opium supply last year; this supply increase may be impacting price as gross income per cultivated hectare decreased 64% and gross family income among opium farmers decreased 56%, based on study interviews [21]. The price per kilogram has decreased in all markets, though prices are markedly different between provinces with lowest prices noted in the northeastern areas [21]. UNODC posits that the declining price may also be due to declining quality (reduced opium content per gram in irrigated fields), competitive lower prices in Tajikistan, and the small number of traders that control the market.

The UNODC survey used satellite images as well as photos and GPS coordinates covering 16% of all arable land in 10 provinces; a survey of farmers was also performed by sampling approximately 8% of all villages in 21 provinces, representing 19% of the total cultivation area [21]. This is the largest study performed to date for estimation of opium cultivation in Afghanistan; however, significant regional differences may not have been adequately assessed in areas under-sampled by the survey. These estimates resulted in a wide confidence interval (109,000–152,000 hectares), though would still represent a 36% increase in cultivation at lowest estimate. Additionally, while opium production is believed to have increased, the study states that production is based on robust estimates as obtaining objective evidence on a crop that is not openly traded is not possible.

This increase in production and the portent of further production increases, indicated by the increasing number of farmers and hectares, has lead Antonio Maria Costa, the executive director of UNODC, to state that,

"Afghan annals will record 2004 as contradictory. Political progress towards democracy culminated in the near plebiscite election of President Karzai. For this splendid accomplishment we all salute President Karzai's courage and determination. Yet, opium cultivation, which has spread like wildfire throughout the country, could ultimately incinerate everything – democracy, reconstruction and stability."[21].

## Current Opium Laws

As the government of Afghanistan develops, laws concerning opium production and use have been the subject of multiple decrees, often with external influence. The United Nations Security Council Resolution and the Bonn Agreement of 2001 stated that the new government of Afghanistan should respect international obligations and cooperate with the international community in the fight against terrorism, drugs and organized crime [22]. In 2002, Hamid Karzai, at the time the appointed interim leader of the Transitional Islamic State of Afghanistan (TISA), issued decrees banning cultivation, production, drug abuse and trafficking of narcotic drugs, and the simultaneous implementation of an eradication campaign by the government [22].

Use of opium products is illegal in Afghanistan; conviction results in a three-month prison sentence.

## Opium Use in Afghanistan

Historically, opium has been used in Afghan communities as medication for different conditions, particularly pain and respiratory complaints. Opium use also has a traditional role in the societies of some groups [23]. There are few national estimates of opium use in Afghanistan; the highest regional use is noted in northeastern Badakhshan Province along the Tajik border, with 20–30% of the local population estimated to be addicted. High use rates have also been reported in districts of Herat and Farah Provinces [23]. In February 2001, UNODC conducted a study in five remote districts of four provinces. The estimated total adult population of these five districts (Khak-e-Jabar, Azro, Hesarak, Gardez, and Sayed Karam) is 120,000 people. According to key informants, there were at least 694 opium users, 164 heroin users, 8514 hashish users and 2556 persons using recreational pharmaceuticals [24]. However, because the interviews were with a limited number of drug users and key informants, these figures are only approximations; there is no official drug user registry in Afghanistan.

Recreational opium use appears to be common in Kabul, based on data from a recent study conducted by UNODC, interviewing 100 key informants and 200 drug users [25]. There are estimated to be at least 6,026 heroin users, 10,257 opium users, 26,415 hashish users, 15,526 pharmaceutical drugs addicts and 8,128 alcohol addicts within Kabul. However, due to the small numbers of drug users interviewed and inherent biases introduced from interview of key informants, these numbers are believed to represent conservative estimates. There are no reports for the number of drug users in other urban areas.

Although heroin is predominately used by men, multiple sources document opiate use starting in childhood and affecting both genders [24,25]. Based on these studies, the Counter Narcotic Department (CND), the highest drug control authority under the presidential office, estimates that there are approximately 500,000 people within Afghanistan addicted to different psychoactive substances (Personal Communication, Dr. M. Zafar, Drug Demand Reduction Officer, CND, October 29, 2004).

Heroin is easily accessible in Afghanistan and there is a disturbing trend towards injection of heroin alone and in combination with other substances, linked to returning refugees importing behaviors from other countries where injection use is common [25,26]. According to a drug user in Kabul: *"Drugs are like vegetables here. Very cheap and infinitely available"*[24]. In Kabul, single use doses of opium cost about 20–50 Afghanis ($0.50–1.00 US) whereas a typical dose of heroin costs about 40–50 Afghanis ($1 U.S.) [26]. However, prices are not stable and change with the seasonal availability of opium and heroin in the local market. Pharmaceutical opiates and other psychoactive substances can be easily obtained from the estimated 15,000 registered pharmacies or many unregistered pharmacies. People can obtain different psychoactive drugs, sedatives, pain killers and narcotics without a prescription and in unlimited quantities [26]. As in Pakistan and India, some pharmacies are reputed to sell buprenorphine (Temgesic) and some addicts report using it, though there is no documented evidence [12,27]. Needles and other injection paraphernalia are available over the counter, but their cost may be prohibitive to drug users who are most often unemployed. Pharmacies are likely to continue as a common source of drugs since the Ministry of Public Health (MOPH) does not currently have the capacity to monitor pharmacies.

Although problem drug use appears to be increasing in Afghanistan, addiction treatment remains limited. Medical services are provided to addicts through both public and private sectors, which, together, are not able to meet the demand for services. In the public sector, the National Mental Health Institutes, under direction of the MOPH, have functioning treatment and rehabilitation centers in several Afghan cities. The center in Kabul (Mental Health Institute) has only 30 treatment slots. (personal communication, Dr. Khaitab Khakar, Director, MoPH Kabul Mental Health Institute, June 30, 2005) In a few provinces, there are branches of the Mental Health Institute providing out-patient services, such as counseling, but these do not have an in-patient facility.

The private sector also has limited treatment resources, with only two non-government organizations (NGO) currently providing in-patient services. The Nejat Center has ten treatment beds and two outreach teams in each of their Kabul and Badakhshan locations. According to the Nejat Center director, Dr. Tareq Suleyman,"We have the capacity to treat just 20 addicts a month but we have 3,000 people on the waiting list "[28]. Between 2001 and 2003, 4335 drug addicts have been treated, with 956 treated at the Kabul Mental Health Institute and 1308 at the Nejat Center [28]. Another NGO, Welfare Association for Afghanistan (WADAN), has a fifteen bed facility for drug addicts in Gardez, Paktiya Province. The standard of care for rehabilitation in Afghanistan is a fifteen day in-patient stay, followed by continued counseling via outreach counselors in the home or return visits to the outpatient department. Methadone treatment has not yet been introduced, though several groups agree that substitution therapy is needed in this setting.

No data is available on relapse due to lack of a reliable, functioning follow-up system. Human resources are scarce for harm reduction activities, like drug demand reduction and rehabilitation, due to lack of trained staff and a severe shortage of female health workers and counselors. There are currently a small number needle exchange programs in Kabul, orchestrated through *Zindagi Nawin* drug counseling programs. (Personal communication, Dr. M. Ilyas Azami, German Technical Cooperation, August 16, 2005) NGO activities involved in harm reduction education are limited, with the majority of their activities conducted in Kabul city, though counseling and prevention activities are being conducted by Nejat in Kabul and German Technical Cooperation (GTZ) with NGO partners SHRO (Herat), Wadan, (Gardez and Kandahar) and KOR in Kabul and Faizabad.

## Regional Opium Use and Influential Trends

The experiences and influence of other countries in the region are an important consideration for predicting future harm reduction needs and blood-borne infection rates in Afghanistan. Larger supplies of heroin are anticipated to be available in Afghanistan as production increases and spillover from new trafficking routes threatens to affect a larger number of people by reaching remote areas of the country.

Data for heroin production within Afghanistan is based on border seizures. Central Asian countries, particularly Tajikistan, are reporting record amounts of drug seized, with the disturbing trend of drug transition from opium to heroin as early as 2001 [16,29]. Security has increased at the Iranian border as a part of that country's response to rising drug use and violence associated with trafficking, but the heroin demand continues in Iran, driving trafficking activity [2,29]. Additionally, trafficking has increased to Central Asia and Pakistan, with the risks of transporting blood-borne pathogens intrinsic to trafficking activities [29]. Traffickers routinely test the quality of the substance with the dealer/distributor in the next country, often sharing injection equipment. These activities allow transmission of infection from areas of presumed higher prevalence to Afghanistan and could initiate or fuel the final component of the cycle related to heroin.

The concern for transmission of blood borne viruses in this context cannot be minimized. Both hepatitis B and C have measurable documented prevalence in injection drug users (IDUs) and the general populations of bordering countries Pakistan and Uzbekistan [3,30-34]. In Pakistan, hepatitis C prevalence ranges from 5.3 to 7% in the general population, [30-32] 22% in non-injecting heroin users,[34] and 89% in IDUs [3]. Rising prevalence of hepatitis B and C due to injection drug use have been noted in other Central Asian Republics [30,35]. Central and South Asia are experiencing a rapid increase in HIV cases introduced by injection drug use and the commercial sex trade [7,16,36-38]. The HIV prevalence among IDUs in neighboring countries is largely unknown. Recently, prevalences of 29.8% and 12.1% were reported among intravenous drug users in Dushanbe, Tajikistan and Tashkent, Uzekistan respectively; of all HIV cases in Iran, 65% are among IDUs [1,39,40]. Injection drug use appears to be increasing in Afghanistan, raising concerns that a concentrated epidemic of HIV will ensue, as IDU and HIV have been documented to follow overland heroin trafficking routes [6,19,41].

The epidemic of injection drug use in Central Asia has been attributed to the poor socioeconomic conditions and proximity to opium trafficking routes [42]. These factors may contribute to the increasing number of IDU in Afghanistan. However, Afghanistan has several other characteristics predisposing its populace to drug addiction and transition to injecting use. Previous studies have documented that refugees are at increased risk to adopt drug use, largely due to poor economic indicators and psychological changes leading to increased risky behavior [43,44]. An estimated 3.5 million Afghans have repatriated within the last four years, of whom a significant proportion remain internally displaced [45]. Two recent studies suggest importation of learned drug use and other risk behaviors by this vulnerable population [34,46]. New behaviors learned by Afghan refugees in Pakistan, and, to a lesser degree, Iran and the Central Asian Republics, where rates of both injection drug use and blood-borne infections are quickly rising, may be impacting drug use patterns [29,37]. Afghans may be disproportionately at risk for blood-borne infections resulting from injection drug use as displaced Afghan drug users exhibited less knowledge regarding HIV transmission and engage in high-risk behavior with greater frequency when compared to Pakistani drug users. A study done among IDU in Quetta, Pakistan revealed that, of 143 Afghans surveyed, none used condoms, only 4% had ever heard of HIV/AIDS, 18% injected drugs, and of those, 72% reported needle sharing, all of which displayed a significantly greater degree of risk than their Pakistani counterparts. Additionally, 41% of Afghan drug users stated they had engaged the services of commercial sex workers [46]. There have been efforts to increase awareness of blood-borne infection transmission among vulnerable groups in Kabul city by several non-government organizations, including ORA, Nejat Center, and GTZ, as well as by the Ministry of Public Health and the National HIV/AIDS Control Program (NACP). The outreach workers affiliated with these programs have established rapport with several marginalized risk groups, predominantly drug users. Preliminary findings from an on-going study of blood-borne infection prevalence among injection drug users in Kabul indicates that, of 67 surveyed, the majority report not sharing "works" and purchasing single use syringes from the pharmacy daily (cost 3 Afghanis = US$0.06). However, another study surveying high-risk and sentinel population groups in Kabul, Heart, Mazar-i-Sharif, and Kandahar notes that only approximately 40% of those surveyed, including drug users, had ever heard of HIV/AIDS. (Personal communication, John Foran, ActionAid Afghanistan, August 16, 2005) Prevention messages have also been disseminated to the general population. The NACP has engaged the religious community in dialogue about the risks of HIV to Afghanistan and their role in community preventive education in a particularly noteworthy program.

There have been few changes in the number or content of rehabilitation programs in Kabul city, though some NGOs wish to initiate substitution therapy following procurement of funding. (Personal communication, Wayne Bazant, German Technical Cooperation, July 6, 2005) UNODC is currently conducting a country-wide assessment of drug use, which may also provide compelling evidence for increasing both the available number and therapeutic options of rehabilitation programs. Additional in-depth studies of risky behavior, particularly before and after the introduction of a harm reduction program, would provide meaningful data.

## Conclusion

Although Afghanistan is a major producer of heroin, injection drug use appears to be a relatively new phenomenon. Greater numbers of heroin users have been observed following the end of the Taliban regime and the return of Afghan refugees from neighboring countries [23]. Although few studies are available, high risk behaviors have been documented among Afghan IDUs along with low HIV/AIDS awareness and virtually no condom use [46]. The growing number of injection drug users, the availability of heroin, and small, geographically-limited number of harm reduction and drug treatment programs in Afghanistan place the country at great risk for epidemics of blood-borne infection. Further research on blood-borne infection risk behaviors and seroprevalence among drug users in Afghanistan would be helpful to better describe the current situation. Funding of programs to broaden education programs on HIV/AIDS and viral hepatitis, harm reduction, and drug treatment services should be an urgent priority.

## Statement of Competing interests

The author(s) declare they have no competing interests.

## Authors' contributions

CT researched and wrote the section on the history of opium cultivation and use in Afghanistan as well as the section on injection drug use trends in Central Asia. NS researched and wrote the section on the current Afghan situation, including law, government policy, and treatment services available. SS researched and wrote the summary statements and contributed to the section on regional influence. All authors read and approved the final manuscript.
